# LEDGF/p75 is critical but not essential for multiple-round HIV 1 replication

**DOI:** 10.1186/1758-2652-13-S3-O10

**Published:** 2010-11-04

**Authors:** RLG Schrijvers, J De Rijck, R Gijsbers, K Ronen, FD Bushman, Z Debyser

**Affiliations:** 1 Molecular Virology and Gene Therapy, KULeuven, Leuven, Belgium; 2 School of Medicine Philadelphia, University of Pennsylvania, Philadelphia, Pennsylvania, PA 19104, USA

## Background

Via its interaction with the cellular cofactor LEDGF/p75, HIV-1 integration is targeted towards active genes. Several strategies were used to show the important role of LEDGF/p75 in viral replication. After RNAi-mediated knockdown of LEDGF/p75, residual replication was observed, possibly supported by minute LEDGF/p75 protein levels. Mouse knockout fibroblasts were generated, enabling analysis of high-titer, single-round lentiviral vector transduction, but not multiple-round replication. To enable evaluation of multiple-round replication in the complete absence of LEDGF/p75, a human LEDGF/p75 knockout cell line was generated (-/-), leaving the p52 splice variant intact.

## Methods

By homologous recombination in Nalm6 cells exons 10 to 13, coding for the LEDGF/p75 integrase binding domain (IBD) were deleted. As a result, a truncated protein was formed in which the C-terminal region of LEDGF/p75 (aa 325-530) was replaced by a new 9 aa tail.

## Results

Correct homologous recombination was verified by southern blot analysis and DNA sequencing. Absence of LEDGF/p75 specific mRNA was verified by Q-PCR. Western blot analysis revealed the presence of the truncated protein. Using 454 sequencing, the HIV integration site profile was determined. In line with data published earlier, integration in the +/+ and +/- cells was favoured in transcription units. In the absence of LEDGF/p75, integration occurs away from genes and a preference for CpG islands emerges. Multiple-round HIV replication in LEDGF/p75 -/- and +/- cells revealed a delayed replication of two weeks.

## Conclusions

Our results corroborate LEDGF/p75 as a critical but not essential cofactor for HIV replication in human cells. We are currently evaluating LEDGF/p75 knockout escape mutants to understand how HIV is capable of replicating in the absence of LEDGF/p75.

